# The Attenuation of Moutan Cortex on Oxidative Stress for Renal Injury in AGEs-Induced Mesangial Cell Dysfunction and Streptozotocin-Induced Diabetic Nephropathy Rats

**DOI:** 10.1155/2014/463815

**Published:** 2014-04-30

**Authors:** Minghua Zhang, Liang Feng, Junfei Gu, Liang Ma, Dong Qin, Chan Wu, Xiaobin Jia

**Affiliations:** ^1^Key Laboratory of New Drug Delivery Systems of Chinese Materia Medica, Jiangsu Provincial Academy of Chinese Medicine, Nanjing, Jiangsu 210028, China; ^2^Department of Pharmacy, Jiangsu University, Zhenjiang, Jiangsu 212013, China; ^3^College of Pharmacy, Nanjing University of Chinese Medicine, Nanjing, Jiangsu 210046, China; ^4^National Center for Toxicological Research, Jefferson, AK 72079, USA

## Abstract

Oxidative stress (OS) has been regarded as one of the major pathogeneses of diabetic nephropathy (DN) through damaging kidney which is associated with renal cells dysfunction. The aim of this study was to investigate whether Moutan Cortex (MC) could protect kidney function against oxidative stress *in vitro* or *in vivo*. The compounds in MC extract were analyzed by HPLC-ESI-MS. High-glucose-fat diet and STZ (30 mg kg^−1^) were used to induce DN rats model, while 200 *μ*g mL^−1^ AGEs were for HBZY-1 mesangial cell damage. The treatment with MC could significantly increase the activity of SOD, glutathione peroxidase (GSH-PX), and catalase (CAT). However, lipid peroxidation malondialdehyde (MDA) was reduced markedly *in vitro* or *in vivo*. Furthermore, MC decreased markedly the levels of blood glucose, serum creatinine, and urine protein in DN rats. Immunohistochemical assay showed that MC downregulated significantly transforming growth factor beta 2 (TGF-*β*2) protein expression in renal tissue. Our data provided evidence to support this fact that MC attenuated OS in AGEs-induced mesangial cell dysfunction and also in high-glucose-fat diet and STZ-induced DN rats.

## 1. Introduction

Diabetic nephropathy (DN), leading to the high morbidity and mortality throughout the world, has been well regarded as one of the common microvascular complications in diabetic patients [1]. It has been well shown that the progress of DN is related to various factors such as glucose metabolism disorders, hypertension, and obesity [2]. A growing body of evidence showed that oxidative stress (OS) played a crucial role in the development and progression of DN [3, 4]. OS, the result of excessive production of reactive oxygen species (ROS) [5], can induce mitochondrial dysfunction, decline adenosine triphosphate, and then lead to DN [6].

Advanced glycation end products (AGEs), playing a central role in DN, are accumulated in glomerular basement membrane, mesangial cells, and endothelial cells [7]. The interaction between AGEs and their specific receptors could trigger OS damage and then lead to signaling cascade events [8–10]. Transforming growth factor beta family (TGF-*β*) was reported to have great relationships with OS via the regulation of protein levels of antioxidant enzymes such as superoxide dismutase (SOD), glutathione peroxidase (GSH-PX), catalase (CAT), and others [11]. Furthermore, plenty of evidences suggested that TGF-*β* was expressed in renal glomeruli, and it usually led to the development of DN [12].

Moutan Cortex (MC), the root bark of* Paeonia suffruticosa* Andr., has been used for treating disease in China and other Asian countries for a long time. It has been shown to have a protective effect against atherosclerosis, infection, inflammation, and other symptoms [13]. It is worthy of note that MC has an inhibitory effect on the production of ROS [14]. However, there are little reports referring to the effect of MC on AGEs-induced oxidative stress* in vitro* and high-glucose-fat diet as well as STZ-induced oxidant damage* in vivo*. The present study aimed to explore the attenuation of MC on AGEs-induced oxidative stress for renal injury in AGEs-induced mesangial cell dysfunction* in vitro* and high-glucose-fat diet as well as STZ-induced DN rats* in vivo*.

## 2. Material and Methods

### 2.1. Chemicals and Reagents

MC, the roots of* Paeonia suffruticosa* Andr. (batch number 20120415), was purchased from Anhui Huqiao Chinese Medicine Technology Co., Ltd. (Tongling, Anhui Province). The pharmaceutical botany of the materia medica was identified by Professor Dekang Wu from Nanjing University of Chinese Medicine. The remaining voucher specimen (number MC20120821) was deposited at Jiangsu Provincial Academy of Chinese Medicine. HPLC-grade acetonitrile was purchased from Burdick & Jackson (Muskegon, MI). HPLC-grade water was obtained using a water purification system (Milli-Q Reagent Water System, MA, USA). Other chemicals for HPLC were of analytical reagent grade. STZ, amino guanidine (AG), vitamin E (VE), bovine serum albumin (BSA), and D-glucose were purchased from Sigma (St. Louis, MO, USA). TGF-*β*2 antibody was from Santa Cruz Biotechnology (Santa Cruz, CA). Rat HBZY-1 mesangial cell line was provided by Wuhan Boster Biological Technology Co., Ltd. (Wuhan, China). In addition, basal DMEM medium and fetal bovine serum (FBS) were provided by Gibco. Total antioxidant capacity assay and ROS kits were purchased from Beyotime Institute of Biotechnology (Nantong, China). SOD, malondialdehyde (MDA), GSH-PX, CAT, glucose measurement, creatinine determination, and urinary protein quantification kits were provided by Jiancheng Bioengineering Institute (Nanjing, China).

### 2.2. Preparation of Extracts of Moutan Cortex

MC (100 g) was refluxed in 500 mL 75% (v/v) ethanol for 30 min. The extraction procedure was repeated twice and all the extracts were combined together. After removing ethanol under reduced pressure, the concentrated extract was diluted to 0.25 g mL^−1^ and stored at 4°C.

### 2.3. Chromatographic Conditions and Equipment

HPLC instrument of Agilent 1100 series (Agilent Technologies, Santa Clara, CA, USA) was performed for MC analysis. The extract was separated on an Agilent XDB-C_18_ column (4.6 mm × 150 mm, 5 *μ*m). The mobile phase gradient conditions consisted of acetonitrile (A) and 0.1% formic acid (B) was 0~20 min, 5~10% A; 20~30 min, 10% A; 30~80 min, 10~18% A; 80~120 min, 18~50% A. The flow rate was 0.8 mL min^−1^, while the column temperature maintained at 25°C. The sample was detected at 254 nm. Sample injection volume was 5 *μ*L.

HPLC-ESI-MS analysis was operated under positive-ion mode. The optimized operating parameters were as follows: ion spray voltage: 4.5 kV; heated capillary temperature: 300°C; capillary voltage: 5 V; auxiliary gas (N_2_) pressure: 10 arbitrary units; sheath gas (N_2_) pressure: 40 arbitrary units. The mass spectrometer was detected over a range of* m/z* 80 to 2000 in the full scan mode.

### 2.4. Preparation of AGEs

AGEs were prepared as previously described [15, 16]. In brief, BSA of 5 g and 9 g D-glucose were dissolved in 100 mL phosphate buffer saline (PBS, 0.2 M, pH = 7.4). After passing through 0.22 *μ*m microporous membrane filter, the solution was incubated under sterile conditions in 5% CO_2_ at 37°C for 3 months. To remove unincorporated glucoses and low molecular reactants, the brown reaction mixture was then dialyzed against 0.01 M PBS overnight. AGEs ELISA kit was used for the measuring of the content of AGEs. The obtained AGEs were stored at 4°C until use.

### 2.5. Cell Culture

Rat mesangial cells (HBZY-1) were maintained in low-glucose DMEM medium (Gibco) with 10% fetal bovine serum (FBS, Gibco). Cells were grown in cell culture dishes and incubated in 5% CO_2_/95% air at 37°C. All media should be replaced every 2 days. When generating the 80–90% confluent layer, the cells were used for the experiment.

### 2.6. Animal Model

Male Sprague Dawley rats weighing 180–220 g were purchased from Shanghai SLAC Laboratory Animal Co. Ltd. (Shanghai, China). The procedures of all animals were in accordance with national and international laws for the use and care of laboratory animals. Actually, all the rats were given distilled water* ad libitum* and kept at a temperature of 25°C and a relative humidity at 45% for three days. DN rats were induced by high-glucose-fat diet (formula: 5% lard, 10% sucrose, 1% cholesterol, 0.2% cholate, 10% egg yolk powder, and 68.8% standard rat feed) for one month and a single intraperitoneal injection of STZ (30 mg kg^−1^ in 0.1 M buffer, pH 4.5) prepared according to the method as described previously [17]. Rats with blood glucose excess of 12 mM and urine protein excess of 20 mg 24 h^−1^ were applied to further study [18]. The rats with normal diet were used as control group. DN rats were randomly divided into 6 groups (*n* = 6/group, Table 1): the model group (DN), positive control groups (treated with AG and VE, 0.1 g kg^−1^, resp.), high dose of MC group (5 g kg^−1^), medium dose of MC group (2.5 g kg^−1^), and low dose of MC group (1.25 g kg^−1^). All rats were administered daily for one month. Blood glucose, serum creatinine, and urine protein were determined by kits according to the manufacturer's protocols at 505 nm, 510 nm, and 595 nm in a microplate reader, respectively.

### 2.7. Immunohistochemical Assay

Rat kidneys were removed under anesthesia and were preserved by perfusion fixation with a solution of 4% paraformaldehyde. After that, tissues were blocked in paraffin and then cut to 5 *μ*m thickness. To retrieve antigens, the sections were heated for 20 min in 10 mM sodium citrate buffer (pH 6.0). According to endogenous peroxidase, slides were incubated in hydrogen peroxide in methanol to reduce nonspecific background staining. Sequentially, tissues were boiled in citrate buffer solution for 10 min. They were cooled and then washed by PBS before the application of blocking serum. Primary antibody anti-TGF-*β*2 (1 : 500) was incubated with tissues and then probed with secondary antibody. Elivison two-step method was performed for the immunohistochemical staining. DM2500 optical microscope was used to collect pictures.

### 2.8. Total Antioxidant Capacity Assay with ABTS Method

ABTS assay was performed to determine the total antioxidant capacity of MC according to the manufacturer's protocols. ABTS working solution and MC of different concentrations (0.05, 0.1, 0.2, 0.4, 0.6, 0.8, and 1.0 mg mL^−1^) were added into 96-well plate. After being incubated at room temperature for 2–6 min, the optical density (OD) value was determined at 734 nm wavelength in a microplate reader. To obtain the standard curve, the total antioxidant capacity of MC could be calculated conveniently.

### 2.9. Reactive Oxygen Species (ROS) Assay

To evaluate intracellular ROS generation, HBZY-1 cells were probed with the redox sensitive dye 2,7-dichlorodihydrofluorescein diacetate (DCFH-DA) in a dark humidified chamber for 20 min at 37°C. At the end of the incubation, PBS was used to wash away the free DCFH-DA molecules. ROS generation was labeled with red fluorescence and visualized by photograph under a fluorescence microscope to calculate the relative fluorescence intensity.

### 2.10. Determination of CAT and GSH-PX

CAT and GSH-PX in this study were performed by CAT kit and GSH-PX kit according to the manufacturer's protocols. The absorbance of samples was determined at 405 nm for CAT and 412 nm for GSH-PX at the end of reaction on a microplate reader.

### 2.11. Determination of SOD and MDA

The cell supernatant and rats serum were collected for the assays of SOD activity and MDA content according to the manufacturer's protocols. In the end of reaction, the optical density (OD) of resultant samples was measured at 550 nm for SOD activity and 450 nm for MDA content in a microplate reader. Both the SOD activities and MDA contents of samples were calculated by the indicated formula: SOD activity (U mL^−1^) = (the absorbance in control group − the absorbance in sample group)/the absorbance in control group/50%; MDA content (nmol mL^−1^) = (the absorbance of sample − the absorbance of standard blank sample/the absorbance of standard sample − the absorbance of standard blank solution) × standard concentration (10 nmol mL^−1^).

### 2.12. Statistical Analysis

All data were taken from three independent experiments and then expressed as means ± standard deviation (SD). One-way ANOVA was performed to compare the statistical analysis by GraphPad Prism 5.0 (San Diego, CA, USA). Tukey's test was then followed to determine the difference between groups. In conclusion, statistical significance was indicated by the *P* value which was less than 0.05.

## 3. Results

### 3.1. Component Analysis of MC

**Figure 1:**
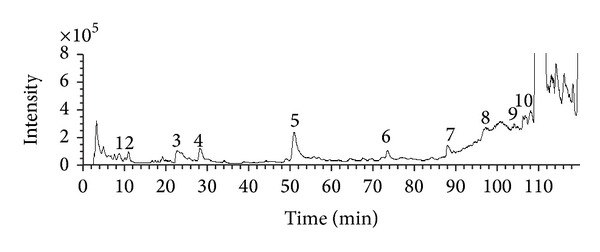
TIC chromatogram of the ethanol extract of MC in positive mode.

**Figure 2:**
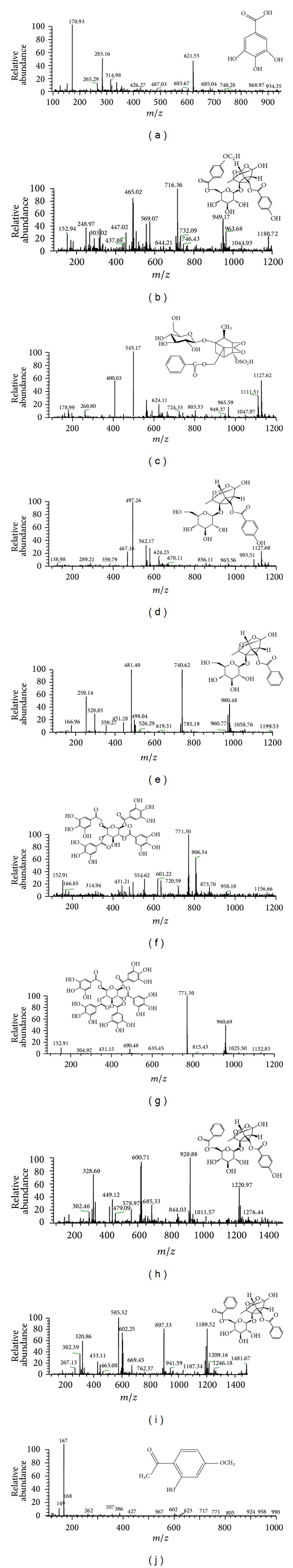
MS spectrum of main peaks.

Phytochemical study has shown that there are lots of chemical compounds containing MC such as paeoniflorin, oxypaeoniflorin, paeonoside, and benzoylpaeoniflorin [19]. In order to identify the main compounds in the extract of MC, HPLC-ESI-MS assay was performed in this experiment. Total ion chromatogram (TIC) of MC was shown in Figure 1. MS spectrums of the main peaks were shown in Figure 2. Hence, according to the chromatographic retention time, relative molecular mass, fragment ion information, relevant literature data, and MS fragmentation ion for each compound, the possible chemical structures were speculated in Table 2.

### 3.2. MC Increased Body Weight and Decreased Kidney Weight of DN Rats

**Figure 3:**
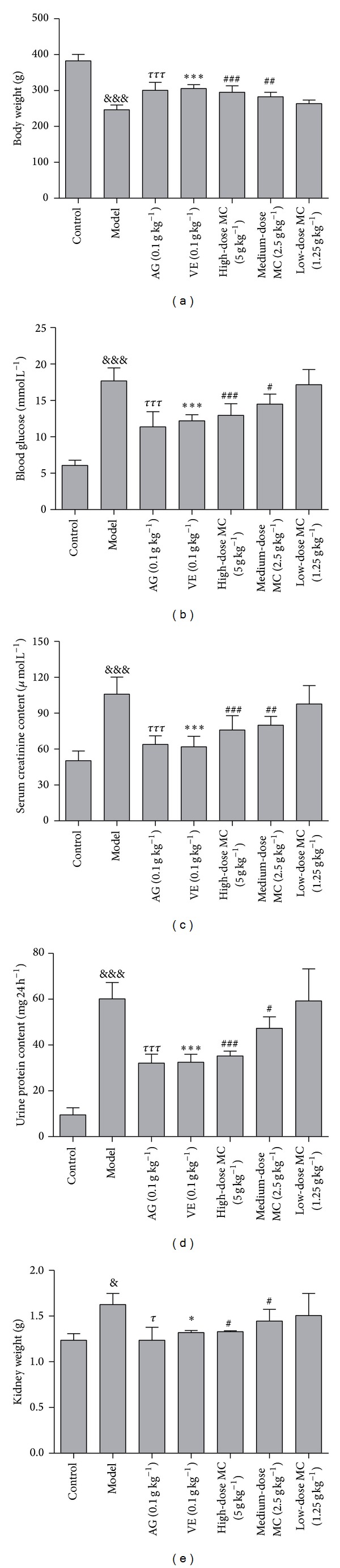
Effect of MC on the body weight loss (a), blood glucose level (b), serum creatinine content (c), and urine protein content (d) in STZ-induced DN rats and kidney weight (e). After being treated with high-glucose-fat diet, following with a single intraperitoneal injection of STZ (30 mg kg^−1^), the rats were treated with MC of high dose (5 g kg^−1^), medium dose (2.5 g kg^−1^), low dose (1.25 g kg^−1^), and positive drugs (AG and VE, 0.1 g kg^−1^, resp.) for 30 days. ^&&&^ *P* < 0.001, model versus blank control; ^*τττ*^ *P* < 0.001, AG versus model; ***P < 0.001, VE versus model; ^###^ *P* < 0.001, ^##^ *P* < 0.01, ^#^ *P* < 0.05, high-dose versus model, medium-dose versus model. Data from individual experiments are presented as means ± SD (*n* = 6).

Abnormal insulin regulation secretion has been proved to be a significant effect on the weight loss of DN rats. As shown in Figure 3(a), the body weight of the rats was decreased significantly compared to the blank control group after injection of STZ (*P* < 0.001, versus blank control). Interestingly, the oral administration of MC at concentrations of 5, 2.5, and 1.25 g kg^−1^ for one month could significantly increase the body weight of DN rats in a dose-dependent manner (*P* < 0.01, versus model).

Relative kidney weight is also one of the major factors determining the severity of nephropathy. As shown in Figure 3(e), compared with control group, DN rats were renal hypertrophy based on the kidney weight. After the treatment with MC, this renal hypertrophy could be ameliorated compared with model DN rats. Hence, our data indicated that MC held a beneficial effect for the treatment of DN.

### 3.3. MC Decreased the Blood Glucose Level in DN Rats

In the present study, the treatment with high-glucose-fat diet and 30 mg kg^−1^ STZ increased significantly the blood glucose level (Figure 3(b)). However, this level was ameliorated significantly by the treatment of AG and VE (*P* < 0.001, versus model), respectively. More importantly, the oral administration of MC dramatically reduced the high blood glucose level, too (*P* < 0.05, *P* < 0.001, versus model). The results suggested that MC could ameliorate the hyperglycemia of DN.

### 3.4. MC Decreased Serum Creatinine and Urine Protein Level in DN Rats

As shown in Figure 3(c), the serum creatinine level was elevated significantly after the injection of STZ in model group (105.92 ± 14.31 *μ*mol L^−1^) (*P* < 0.001, versus blank control). However, this high level was decreased remarkably by the treatment of AG (63.99 ± 7.08 *μ*mol L^−1^, *P* < 0.001, versus model) and VE (58.57 ± 3.68 *μ*mol L^−1^, *P* < 0.001, versus model). Similarly, the treatment with MC decreased significantly the serum creatinine level in DN rats (*P* < 0.01, versus model). These findings indicated that MC might prevent the accumulation of serum creatinine and improve the renal function.

In the present study, the treatment of high-glucose-fat diet and STZ significantly increased urine protein level (60.19 ± 7.10 mg 24 h^−1^, *P* < 0.001, versus blank control). The amelioration of MC on urine protein symptom was shown in Figure 3(d). MC decreased significantly the urine protein level in DN rats (35.20 ± 2.19 mg 24 h^−1^ for high-dose, *P* < 0.001, versus model; 47.29 ± 5.02 mg 24 h^−1^ for medium-dose, *P* < 0.05, versus model). From what has been discussed above, we conclude that MC holds an effect on reducing the serum creatinine and urine protein level in renal injury.

### 3.5. MC Downregulated TGF-*β*2 Protein Expression in Kidney Tissue

**Figure 4:**
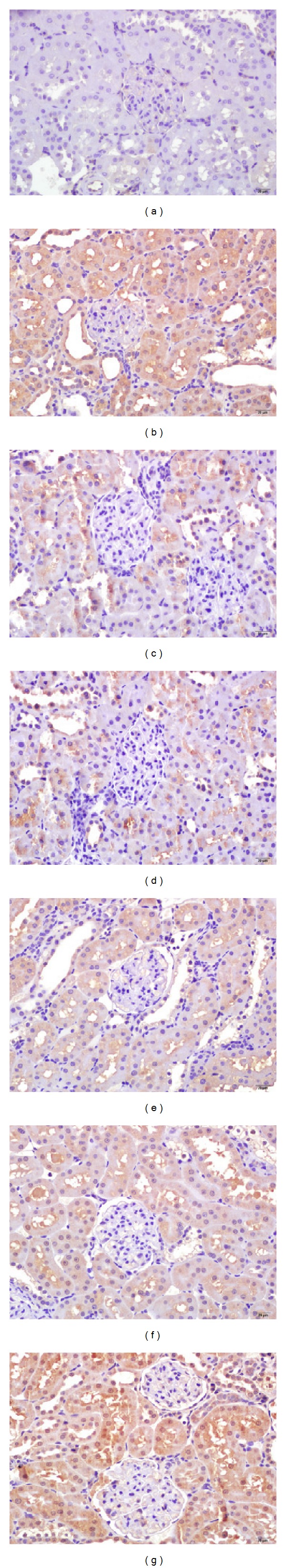
The downregulation of MC on STZ-induced TGF-*β*2 protein expression in kidney production. (a) Control blank; (b) model group (STZ); (c) AG group (0.1 g kg^−1^); (d) VE group (0.1 g kg^−1^); (e) MC (high-dose, 5 g kg^−1^); (f) MC (medium-dose, 2.5 g kg^−1^); (g) MC (low-dose, 1.25 g kg^−1^).

As shown in Figure 4, the expression of TGF-*β*2 in DN model rats was significantly enhanced compared to the control blank rats. However, after the treatment with MC for 30 days, the overexpression of TGF-*β*2 protein illustrated as brown staining was attenuated significantly in a dose-dependent manner compared with model group. Our experimental results suggested that MC could ameliorate renal damage via downregulating TGF-*β*2 protein expression.

### 3.6. Total Antioxidant Capacity of MC

**Figure 5:**
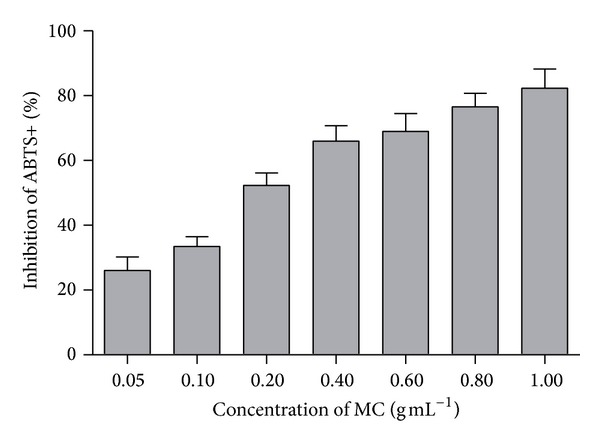
The inhibition of MC on ABTS^+^ generation. Data from individual experiments are presented as means ± SD (*n* = 3).

In order to explore the antioxidant capacity of MC, we tested the total antioxidant capacity of MC using total antioxidant capacity assay kit with ABTS method. As shown in Figure 5, the inhibitory effects of MC at the concentrations of 0.05, 0.1, 0.2, 0.4, 0.6, 0.8, and 1.0 mg mL^−1^ were 26.02 ± 10.34%, 33.46 ± 7.38%, 52.30 ± 9.30%, 65.98 ± 11.50%, 68.92 ± 13.51, 76.50 ± 10.36, and 82.29 ± 14.47%, respectively. The IC_50_ of MC on inhibiting ABTS^+^ generation was 0.19 mg mL^−1^. These findings demonstrated that MC had a great antioxidant capacity.

### 3.7. MC Attenuated AGEs-Induced ROS Generation in HBZY-1 Mesangial Cell

**Figure 6:**
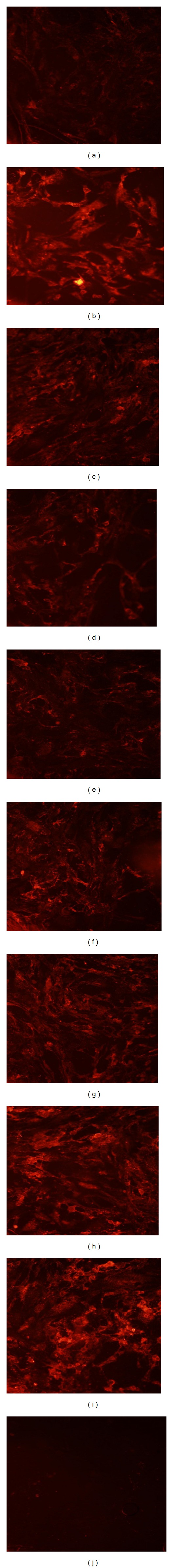
Attenuation of MC on AGEs-induced ROS generation in HBZY-1 mesangial cell. (a) BSA (200 *μ*g mL^−1^); (b) AGEs (200 *μ*g mL^−1^); (c) AG (10 *μ*M) + AGEs (200 *μ*g mL^−1^); (d) VE (10 *μ*M) + AGEs (200 *μ*g mL^−1^); (e) MC (2 × 10^−4^ g mL^−1^) + AGEs (200 *μ*g mL^−1^); (f) MC (10^−4^ g mL^−1^) + AGEs (200 *μ*g mL^−1^); (g) MC (5 × 10^−5^ g mL^−1^) + AGEs (200 *μ*g mL^−1^); (h) MC (2.5 × 10^−5^ g mL^−1^) + AGEs (200 *μ*g mL^−1^); (i) MC (1.25 × 10^−5^ g mL^−1^) + AGEs (200 *μ*g mL^−1^); (j) negative.

As depicted in Figure 6, the fluorescence intensity in HBZY-1 mesangial cell was enhanced significantly after the treatment with 200 *μ*g/mL AGEs compared to 200 *μ*g mL^−1^ BSA. However, the overgeneration of ROS was reduced markedly by the treatment with MC (1.25 × 10^−5^ g mL^−1^, 2.5 × 10^−5^ g mL^−1^, 5.0 × 10^−5^ g mL^−1^, 1.0 × 10^−4^ g mL^−1^, and 2.0 × 10^−4^ g mL^−1^). Our findings demonstrated that MC could attenuate AGEs-induced intracellular ROS overgeneration in HBZY-1 mesangial cell.

### 3.8. MC Increased CAT Activity and GSH-PX Activity in HBZY-1 Mesangial Cell and the Serum of DN Rats

**Figure 7:**
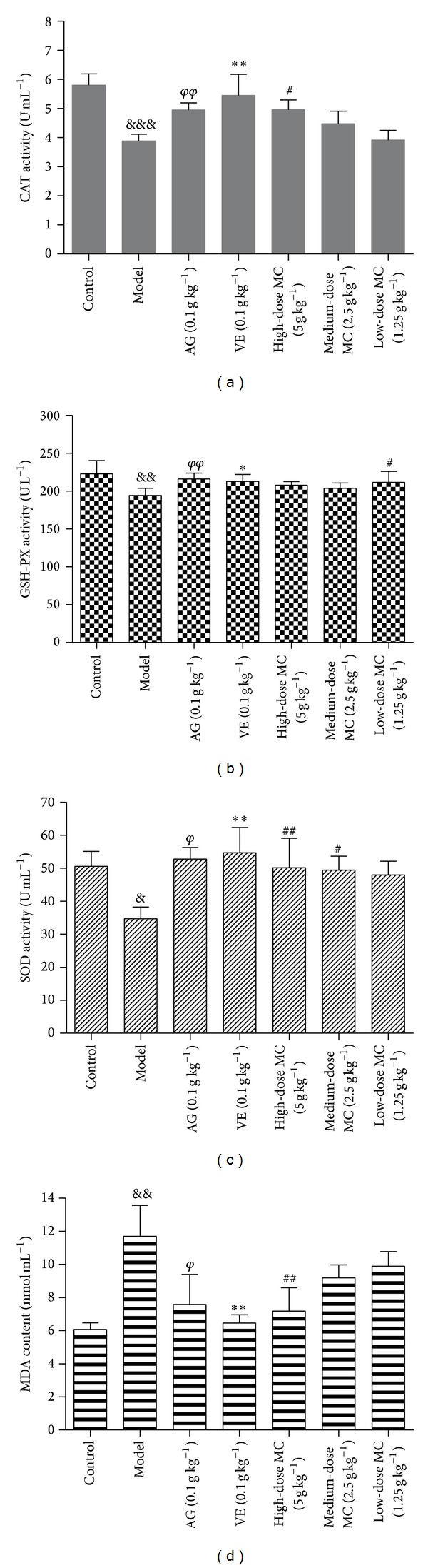
Effect of MC on STZ-induced oxidant stress in DN rats. (a) The effect of MC on STZ-induced CAT activity; (b) the effect of MC on STZ-induced GSH-Px activity; (c) the effect of MC on STZ-induced SOD activity; (d) the effect of MC on STZ-induced MDA content. ^&&&^ *P* < 0.001, ^&&^ *P* < 0.01, and ^&^ *P* < 0.05, model versus blank control; ^*φφ*^ *P* < 0.01 and ^*φ*^ *P* < 0.05, AG versus model; **P < 0.01 and *P < 0.05, VE versus model; ^##^ *P* < 0.01 and ^#^ *P* < 0.05, high-dose versus model, medium-dose versus model, low-dose versus model. Data from individual experiments are presented as means ± SD (*n* = 6).

**Figure 8:**
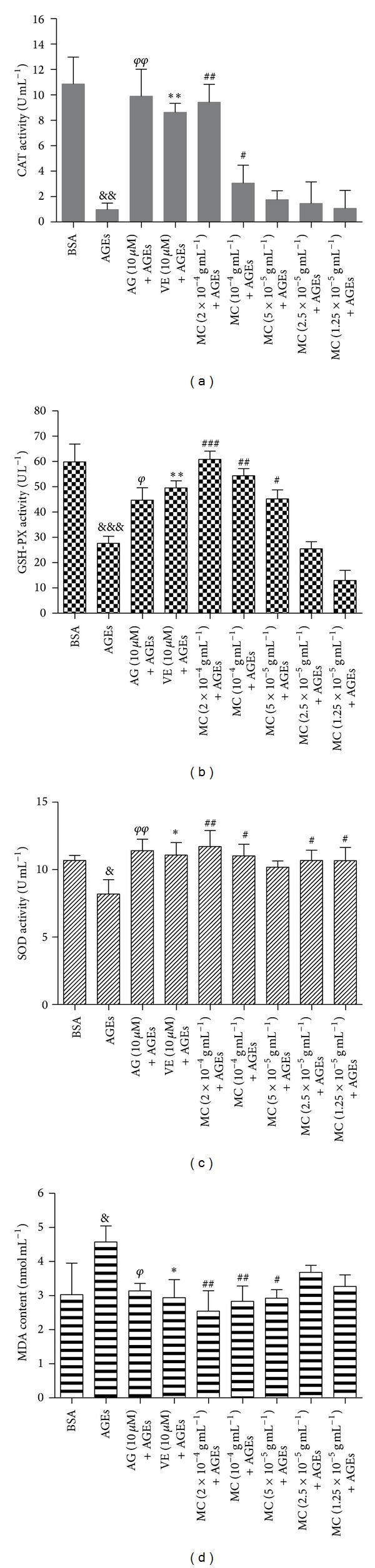
Effect of MC on AGEs-induced oxidant stress in HBZY-1 mesangial cell. (a) The effect of MC on AGEs-induced CAT activity; (b) the effect of MC on AGE-induced GSH-Px activity; (c) the effect of MC on AGEs-induced SOD activity; (d) the effect of MC on AGEs-induced MDA content. ^&&&^ *P* < 0.001, ^&&^ *P* < 0.01, and ^&^ *P* < 0.05, AGEs versus BSA; ^*φφ*^ *P* < 0.01 and ^*φ*^ *P* < 0.05, AG versus AGEs; **P < 0.01 and *P < 0.05, VE versus AGEs; ^###^ *P* < 0.001, ^##^ *P* < 0.01, ^#^ *P* < 0.05, and 2 × 10^−4^ g mL^−1^ versus model, 10^−4^ g mL^−1^ versus model. 5 × 10^−5^ g mL^−1^ versus model, 2.5 × 10^−5^ g mL^−1^ versus model, and 1.25 × 10^−5^ g mL^−1^ versus model. Data from individual experiments are presented as means ± SD (*n* = 3).

CAT and GSH-PX activity in mesangial cell and the serum of DN rats were conducted to evaluate the antioxidant activity of MC on oxidative stress for renal injury. As shown in Figures 7(a)-7(b) and 8(a)-8(b), the CAT and GSH-PX activities in cells or DN rats serum were decreased significantly by exposure to 200 *μ*g/mL AGEs or 30 mg kg^−1^ STZ (*P* < 0.05, *P* < 0.01). However, positive drugs AG (0.1 g kg^−1^) and VE (0.1 g kg^−1^) could increase antioxidant enzymes' activity. As we expected, the treatment with MC (1.25 × 10^−5^ g mL^−1^, 2.5 × 10^−5^ g mL^−1^, 5.0 × 10^−5^ g mL^−1^, 1.0 × 10^−4^ g mL^−1^, and 2.0 × 10^−4^ g mL^−1^ for cell, while 1.25 g kg^−1^, 2.5 g kg^−1^, and 5.0 g kg^−1^ for animal) significantly increased CAT and GSH-PX activities compared with model group (*P* < 0.001). The above data indicated that MC had a potential capacity on attenuating AGEs or STZ-induced oxidant damage for renal injury both* in vivo* and* in vitro*.

### 3.9. MC Increased SOD Activity and Decreased MDA Level in HBZY-1 Mesangial Cell and the Serum of DN Rats

In the present study, the SOD activity and MDA level in cell supernatant and the serum of DN rats were evaluated. As shown in Figures 7(c)-7(d) and 8(c)-8(d), the SOD activity was decreased markedly by 200 *μ*g mL^−1^ AGEs* in vitro* or 30 mg kg^−1^ STZ* in vivo,* while MDA content was increased (*P* < 0.05, versus control). However, the treatment with MC could enhance significantly SOD activity while reduce markedly MDA content in cell supernatant as well as in the serum of DN rats in a concentration-dependent manner (*P* < 0.05, versus AGEs). These data suggested that MC protected HBZY-1 mesangial cell and DN rats from renal injury by attenuating oxidative damage.

## 4. Discussion and Conclusions

DN is one of the microvascular complications of diabetes. Reports have shown that the progress of DN is related to various factors such as glucose metabolism disorders, hypertension, and obesity [2]. Nowadays, accumulating evidences show that abnormal insulin regulation secretion has been proved to be a significant effect on the weight loss of DN patients. Hyperglycemia has been demonstrated to be the key contributor in the development of DN in both type 1 and type 2 diabetes [20, 21]. Hence, hyperglycemia is the most common feature on the diagnosis of diabetes and its complication [22]. Reporters have also shown that early alterations in DN could induce the development of glomerular hyperfiltration, followed by the increased urinary albumin excretion [23]. The increasing protein excretion in urine has been regarded as the major index in DN patients [24]. In addition, the kidney damage and the accumulation of creatinine were important factors in the progression of DN. Therefore, blood glucose, body weight, kidney weight, urinary albumin excretion, and serum creatinine were measured in DN rats in the present study. We observed that the body weight was significantly reduced, whereas kidney weight, blood glucose, urinary albumin excretion, and serum creatinine level were increased in DN rats. Pretreatment with MC significantly reversed the change in DN rats. Hence, we evaluated that MC held a beneficial effect for the treatment of DN.

AGEs, contributors to diabetic microvascular complications which formed through a series of reactions from Schiff bases and Amadori products to stable irreversible end products, were shown to be involved in the pathogenesis DN [25, 26]. Nowadays, accumulating evidence showed that hyperglycemia could mediate the alteration of extra and intracellular metabolism, such as the function of AGEs. The high level of OS associated with cardiovascular disease was linked to prooxidants such as AGEs [27]. AGEs have been regarded as one of the most toxic substances and resulted in OS response in diabetic vascular dysfunction [28–30]. Clinical study also showed that the accumulation of AGEs might contribute to the increase of OS in renal tissue which then leads to DN. OS has been regarded as one of the mechanisms on renal structural and functional alterations, for example, interstitial fibrosis, fibrotic glomeruli, tubular atrophy, and mesangial expansion [31]. Thus, the increase of antioxidant capacity might be one of the important events in the protection of oxidative stress for renal injury. Excitedly, in our study, we observed that MC had a great antioxidant capacity throughout the result of ABTS method. Therefore, MC might have the effect on antioxidant capacity on DN.

According to the reports published, we could observe that AGEs could result in oxidative damage by triggering OS, leading to the overgeneration of ROS [32–34]. It has been demonstrated in several studies that many renal cell types, for example, mesangial cells and endothelial cells, were found to be the producer of high levels of ROS under hyperglycemic conditions [35–37]. Hence, the inhibition of ROS overgeneration seemed to be one of the effective ways in attenuating AGEs-induced oxidative damage. Consistent with previous reports, we observed that the fluorescence intensity in HBZY-1 mesangial cell was enhanced significantly after the treatment with 200 *μ*g mL^−1^ AGEs compared to 200 *μ*g mL^−1^ BSA. However, the overgeneration of ROS was reduced markedly by the treatment with MC. Our findings demonstrated that MC could attenuate AGEs-induced intracellular ROS overgeneration in HBZY-1 mesangial cell.

It has been well defined that AGEs might break the antioxidant defense system via regulating antioxidant enzymes' activity, such as CAT and GSH-PX. Actually, CAT and GSH-PX, two important antioxidases, play important roles in the antioxidant defense system in DN. Many studies have shown that CAT and GSH-PX are able to strengthen the oxidation resistance as the main biochemical target [38–40]. They could also maintain the low steady-state concentration of ROS [41]. In this present study, CAT and GSH-PX activity in mesangial cell and the serum of DN rats were conducted to evaluate the antioxidant activity of MC on oxidative stress for renal injury. Our data demonstrated that MC could increase CAT and GSH-PX activities in HBZY-1 mesangial cells, as well as in serum of DN rats. MC had a potential capacity on attenuating AGEs or STZ-induced oxidant damage for renal injury both* in vivo *and* in vitro*. The protective effect of MC on pathological changes of renal injury in DN might be associated with its function on oxidant damage.

SOD, the cytoprotective antioxidant enzyme, could convert superoxide to hydrogen peroxide to prevent oxidation. The overexpression of SOD exclusively appeared at the accumulation of glucose-induced ROS and the formation of AGEs [42]. MDA, a cell membrane lipid peroxidation product, was used as an indicator of oxidative damage. The lipid peroxidation represented the most frequent injury resulting from the activation of ROS [43, 44]. In the present study, the SOD activity and MDA level in cell supernatant and the serum of DN rats were evaluated. Experiments both* in vitro* and* in vivo* showed that the treatment with MC could attenuate AGEs-induced oxidative damage in HBZY-1 cell and high-glucose-fat diet and STZ-induced oxidative damage in DN rats through reducing the production of lipid peroxidation MDA and increasing SOD activity. These data suggested that MC protected HBZY-1 mesangial cell and DN rats from renal injury by attenuating oxidative damage.

Renal injury had been a key pathological damage in DN progression. Specifically, OS in the kidney could modulate renal hemodynamic actions and alter glomerular permeability, leading to the progression of renal disease [45]. TGF-*β* (containing TGF-*β*1, TGF-*β*2,etc.), a kind of multifunctional cytokine, has been found to affect the adhesion, differentiation and OS of cells, and the cell cycle [46]. Plenty of evidences also demonstrated that TGF-*β* played a crucial role in the development of kidney fibrosis [47, 48]. TGF-*β* could contribute to the renal damage in animal models. It had been well defined that TGF-*β* had a close relationship with OS and it played significant role in the progression of renal disease [11]. The reduction of TGF-*β* expression could significantly regulate OS. Our study in the past has shown that the downexpression of TGF-*β*1 had a protective effect on renal injury in DN [49]. Hence, in the present study, we choose to study the expression of TGF-*β*2. Finally, we could observe that the regulation of MC on oxidative stress had a protective effect on renal injury in DN through AGEs-induced mesangial cell dysfunction and STZ-induced DN* in vivo* and* in vitro*. Our experimental results suggested that MC could ameliorate renal damage via downregulating TGF-*β*2 protein expression.

Overall, the extract of MC could protect the kidney function via decreasing the blood glucose and fibrosis-related factor TGF-*β* expression and improving the serum creatinine, urine protein in DN rats. Moreover, MC could attenuate oxidative stress for renal injury in AGEs-induced mesangial cell dysfunction and high-glucose-fat diet as well as STZ-induced diabetic nephropathy rats through increasing CAT, GSH-PX, and SOD activity detection and decreasing MDA level. Our present study demonstrated that the protective effect of MC on renal injury in DN was associated with its antioxidant activity. Thus, MC might be a beneficial agent for the prevention and treatment of renal injury.

## Figures and Tables

**Table 1:** Definition of rats experimental groups (*n* = 6/group).

No.	Group	kinds of rats	Drugs	Dose (g kg^−1^)
1	Control	Normal	Normal saline	0.1
2	Model	DN	Normal saline	0.1
3	Positive control (1)	DN	AG	0.1
4	Positive control (2)	DN	VE	0.1
5	High dose	DN	Moutan Cortex	5
6	Medium dose	DN	Moutan Cortex	2.5
7	Low dose	DN	Moutan Cortex	1.25

**Table 2:** MS fragmentation ion of the main compounds in the extract of MC.

No.	*t*_*R*_/min	[M + H]^+^ or [M + Na]^+^	Fragment (*m*/*z*)	Compound
1	9.38	171	170, 125	Gallic acid
2	11.61	465	464, 303, 171, 139	Mudanoside B
3	23.08	545	544, 499, 423, 383, 377	Paeoniflorin sulfonate
4	29.64	497	467, 360, 335, 167, 139	Oxypaeoniflorin
5	51.65	481	527, 451, 329, 167, 123	Paeoniflorin
6	73.54	771	601, 305, 431, 233	1,2,3,6-Tetra-O-galloyl-beta-d-glucose
7	88.52	771	601, 431, 305, 279, 261, 413, 449, 233	1,2,3,4,6-Penta-O-galloyl-D-glucopyranose
8	98.42	601	479, 449, 431, 123	Benzoyloxypaeoniflorin
9	104.59	585	555, 463, 433, 123	Benzoylpaeoniflorin
10	108.73	167	149, 125	Paeonol

## References

[1] Fox CS, Larson MG, Leip EP, Meigs JB, Wilson PWF, Levy D (2005). Glycemic status and development of kidney disease: the Framingham Heart Study. *Diabetes Care*.

[2] Hendig D, Tarnow L, Kuhn J, Kleesiek K, Götting C (2008). Identification of a xylosyltransferase II gene haplotype marker for diabetic nephropathy in type 1 diabetes. *Clinica Chimica Acta*.

[3] Thuraisingham RC, Nott CA, Dodd SM, Yaqoob MM (2000). Increased nitrotyrosine staining in kidneys from patients with diabetic nephropathy. *Kidney International*.

[4] Onozato ML, Tojo A, Goto A, Fujita T, Wilcox CS (2002). Oxidative stress and nitric oxide synthase in rat diabetic nephropathy: effects of ACEI and ARB. *Kidney International*.

[5] Maiese K (2008). Diabetic stress: new triumphs and challenges to maintain vascular longevity. *Expert Review of Cardiovascular Therapy*.

[6] Hosseini A, Sharifzadeh M, Rezayat SM (2010). Benefit of magnesium-25 carrying porphyrin-fullerene nanoparticles in experimental diabetic neuropathy. *International Journal of Nanomedicine*.

[7] Fukami K, Yamagishi S-I, Ueda S, Okuda S (2008). Role of AGEs in diabetic nephropathy. *Current Pharmaceutical Design*.

[8] Yamagishi S-I, Imaizumi T (2005). Diabetic vascular complications: pathophysiology, biochemical basis and potential therapeutic strategy. *Current Pharmaceutical Design*.

[9] Yamagishi S-I, Matsui T, Nakamura K (2007). Kinetics, role and therapeutic implications of endogenous soluble form of receptor for advanced glycation and products (sRAGE) in diabetes. *Current Drug Targets*.

[10] Yamagishi S-I, Nakamura K, Matsui T (2009). Regulation of advanced glycation end product (AGE)-receptor (RAGE) system by PPAR-gamma agonists and its implication in cardiovascular disease. *Pharmacological Research*.

[11] Martinez-Palacian A, del Castillo G, Suarez-Causado A (2013). Mouse hepatic oval cells require Met-dependent PI3K to impair TGF-beta-induced oxidative stress and apoptosis. *PLoS ONE*.

[12] Huang J, Matavelli LC, Siragy HM (2011). Renal (pro)renin receptor contributes to development of diabetic kidney disease through transforming growth factor-*β*1—connective tissue growth factor signalling cascade. *Clinical and Experimental Pharmacology and Physiology*.

[13] Wu M, Gu Z (2009). Screening of bioactive compounds from Moutan Cortex and their anti-inflammatory activities in rat synoviocytes. *Evidence-Based Complementary and Alternative Medicine*.

[14] Rho S, Chung H-S, Kang M (2005). Inhibition of production of reactive oxygen species and gene expression profile by treatment of ethanol extract of Moutan Cortex Radicis in oxidative stressed PC12 cells. *Biological and Pharmaceutical Bulletin*.

[15] Li Y, Liu S, Zhang Z (2012). Rage mediates accelerated diabetic vein graft atherosclerosis induced by combined mechanical stress and ages via synergistic erk activation. *PLoS ONE*.

[16] Kim JY, Park HK, Yoon JS (2008). Advanced glycation end product (AGE)-induced proliferation of HEL cells via receptor for AGE-related signal pathways. *International Journal of Oncology*.

[17] Zhong S-Z, Ge Q-H, Qu R, Li Q, Ma S-P (2009). Paeonol attenuates neurotoxicity and ameliorates cognitive impairment induced by d-galactose in ICR mice. *Journal of the Neurological Sciences*.

[18] Li H, Zheng X, Wang H, Zhang Y, Xin H, Chen X (2010). XLF-III-43, a novel coumarin-aspirin compound, prevents diabetic nephropathy in rats via inhibiting advanced glycation end products. *European Journal of Pharmacology*.

[19] Chen G, Zhang L, Zhu Y (2006). Determination of glycosides and sugars in Moutan Cortex by capillary electrophoresis with electrochemical detection. *Journal of Pharmaceutical and Biomedical Analysis*.

[20] Cooper ME (2001). Interaction of metabolic and haemodynamic factors in mediating experimental diabetic nephropathy. *Diabetologia*.

[21] Ha H, Lee HB (2000). Reactive oxygen species as glucose signaling molecules in mesangial cells cultured under high glucose. *Kidney International*.

[22] Lasaridis AN, Sarafidis PA (2003). Diabetic nephropathy and antihypertensive treatment: what are the lessons from clinical trials?. *American Journal of Hypertension*.

[23] Tian S, Tang J, Liu H (2012). Propyl gallate plays a nephroprotective role in early stage of diabetic nephropathy associated with suppression of glomerular endothelial cell proliferation and angiogenesis. *Experimental Diabetes Research*.

[24] Mundel P, Reiser J (2010). Proteinuria: an enzymatic disease of the podocyte. *Kidney International*.

[25] Zhou X, Wang B, Zhu L, Hao S (2012). A novel improved therapy strategy for diabetic nephropathy: targeting AGEs. *Organogenesis*.

[26] Ramasamy R, Yan SF, Schmidt AM (2011). Receptor for AGE (RAGE): signaling mechanisms in the pathogenesis of diabetes and its complications. *Annals of the New York Academy of Sciences*.

[27] Torreggiani M, Liu H, Wu J (2009). Advanced glycation end product receptor-1 transgenic mice are resistant to inflammation, oxidative stress, and post-injury intimal hyperplasia. *American Journal of Pathology*.

[28] Han S-H, Kim YH, Mook-Jung I (2011). RAGE: the beneficial and deleterious effects by diverse mechanisms of actions. *Molecules and Cells*.

[29] Kim W, Hudson BI, Moser B (2005). Receptor for advanced glycation end products and its ligands: a journey from the complications of diabetes to its pathogenesis. *Annals of the New York Academy of Sciences*.

[30] Park L, Raman KG, Lee KJ (1998). Suppression of accelerated diabetic atherosclerosis by the soluble receptor for advanced glycation endproducts. *Nature Medicine*.

[31] Tapia E, Zatarain-Barron ZL, Hernandez-Pando R (2013). Curcumin reverses glomerular hemodynamic alterations and oxidant stress in 5/6 nephrectomized rats. *Phytomedicine*.

[32] Brownlee M (2007). Preventing kidney cell suicide. *Nature Medicine*.

[33] Giacco F, Brownlee M (2010). Oxidative stress and diabetic complications. *Circulation Research*.

[34] Ramasamy R, Vannucci SJ, Yan SSD, Herold K, Yan SF, Schmidt AM (2005). Advanced glycation end products and RAGE: a common thread in aging, diabetes, neurodegeneration, and inflammation. *Glycobiology*.

[35] Kiritoshi S, Nishikawa T, Sonoda K (2003). Reactive oxygen species from mitochondria induce cyclooxygenase-2 gene expression in human mesangial cells: potential role in diabetic nephropathy. *Diabetes*.

[36] Eun AL, Ji YS, Jiang Z (2005). Reactive oxygen species mediate high glucose-induced plasminogen activator inhibitor-1 up-regulation in mesangial cells and in diabetic kidney. *Kidney International*.

[37] Thallas-Bonke V, Thorpe SR, Coughlan MT (2008). Inhibition of NADPH oxidase prevents advanced glycation end product-mediated damage in diabetic nephropathy through a protein kinase C-*α*-dependent pathway. *Diabetes*.

[38] Gupta V, Lahiri SS, Sultana S, Tulsawani RK, Kumar R (2010). Anti-oxidative effect of Rhodiola imbricata root extract in rats during cold, hypoxia and restraint (C-H-R) exposure and post-stress recovery. *Food and Chemical Toxicology*.

[39] Ku S-K, Seo B-I, Park J-H (2009). Effect of Lonicerae Flos extracts on reflux esophagitis with antioxidant activity. *World Journal of Gastroenterology*.

[40] Salo DC, Lin SW, Pacifici RE, Davies KJA (1988). Superoxide dismutase is preferentially degraded by a proteolytic system from red blood cells following oxidative modification by hydrogen peroxide. *Free Radical Biology and Medicine*.

[41] Zamocky M, Furtmüller PG, Obinger C (2008). Evolution of catalases from bacteria to humans. *Antioxidants and Redox Signaling*.

[42] Nishikawa T, Edelstein D, Du XL (2000). Normalizing mitochondrial superoxide production blocks three pathways of hyperglycaemic damage. *Nature*.

[43] Aikemu A, Yusup A, Umar A (2012). The impact of the Uighur medicine abnormal savda munziq on antitumor and antioxidant activity in a S180 and Ehrlich ascites carcinoma mouse tumor model. *Pharmacognosy Magazine*.

[44] Catalá A (2009). Lipid peroxidation of membrane phospholipids generates hydroxy-alkenals and oxidized phospholipids active in physiological and/or pathological conditions. *Chemistry and Physics of Lipids*.

[45] Liu Y, Bledsoe G, Hagiwara M (2010). Blockade of endogenous tissue kallikrein aggravates renal injury by enhancing oxidative stress and inhibiting matrix degradation. *American Journal of Physiology. Renal Physiology*.

[46] Liu R-M, Gaston Pravia KA (2010). Oxidative stress and glutathione in TGF-*β*-mediated fibrogenesis. *Free Radical Biology and Medicine*.

[47] Langham RG, Kelly DJ, Gow RM (2006). Transforming growth Factor-*β* in human diabetic nephropathy: effects of ACE inhibition. *Diabetes Care*.

[48] García-Sánchez O, López-Hernández FJ, López-Novoa JM (2010). An integrative view on the role of TGF-beta in the progressive tubular deletion associated with chronic kidney disease. *Kidney international*.

[49] Zhang MH, Feng L, Zhu MM (2014). The anti-inflammation effect of Moutan Cortex on advanced glycation end products-induced rat mesangial cells dysfunction and High-glucose-fat diet and streptozotocin-induced diabetic nephropathy rats. *Journal of Ethnopharmacology*.

